# Diagnostic value of shear wave elastography for diabetic peripheral neuropathy: comparison between junior radiologists and senior radiologists

**DOI:** 10.1186/s12880-025-02061-w

**Published:** 2025-12-29

**Authors:** Rong-li Peng, Yan-feng Jiang, Hua-liang Shen, Di-jia Ni, Ying Zhou, Xia-tian Liu, Zhen-zhen Jiang

**Affiliations:** Department of Ultrasound, Shaoxing People’s Hospital, The First Affiliated Hospital of Shaoxing University, 568 N Zhongxing Rd, Shaoxing, 312000 China

**Keywords:** Diabetic peripheral neuropathy, Ultrasound, Shear wave elastography, Diagnostic accuracy, Ultrasonography

## Abstract

**Background:**

Diabetic peripheral neuropathy (DPN) is a prevalent complication of diabetes mellitus, and is often underdiagnosed because of its variable clinical presentation and operator–dependent diagnostic tools. Shear wave elastography (SWE), which quantitatively evaluates tissue stiffness, has the potential to enhance conventional ultrasound by improving diagnostic accuracy and consistency. Nevertheless, a comprehensive analysis examining the extent to which the integration of SWE with conventional ultrasound can enhance the diagnostic performance of radiologists across varying levels of expertise has yet to be performed.

**Methods:**

In this study, a total of 458 lower extremities from patients with type 2 diabetes were examined via ultrasound and SWE. Four radiologists (two seniors and two juniors) independently assessed the grayscale ultrasound, SWE, and combined images. Diagnostic performance was compared via receiver operating characteristic (ROC) curves and sensitivity and specificity metrics.

**Results:**

SWE measurements revealed significantly greater stiffness of the tibial nerve in the DPN group than in the non-DPN group, with values of 37.30 kPa versus 25.40 kPa (*P* < 0.001) and corresponding shear wave velocities of 3.54 m/s versus 2.90 m/s (*P* < 0.001). The combined images improved diagnostic accuracy across all readers. Notably, junior radiologists exhibited a substantial improvement in terms of sensitivity (ΔSensitivity = 25.565, 95% CI: 18.477–32.653, *P* = 0.004). In contrast, for the senior radiologists, neither the sensitivity nor the specificity significantly increased with increasing integration SWE.

**Conclusion:**

Combining SWE with conventional ultrasound improves the diagnostic accuracy for DPN and helps reduce performance gaps between junior and senior radiologists. SWE may serve as an effective adjunct to support early detection and consistent evaluation of DPN in clinical practice.

**Supplementary Information:**

The online version contains supplementary material available at 10.1186/s12880-025-02061-w.

## Introduction

Diabetic peripheral neuropathy (DPN) is one of the most common complications of diabetes, affecting more than 50% of diabetic patients. It typically begins with small–fibre damage causing pain and sensory loss, progressing to large–fibre involvement, numbness, and loss of protective sensation [1, 2]. These changes increase the risk of foot ulcers and lower–limb amputations, making early diagnosis critical to prevent serious outcomes.

In clinical practice, DPN is assessed via medical history, physical examination, and simple bedside tests, such as vibration perception, temperature discrimination, and the use of a 10 g monofilament. Various screening tools, including the Michigan Neuropathy Screening Instrument [3], and the Toronto Clinical Scoring System [4], are also available. Nevertheless, up to 50% of DPN patients may remain asymptomatic [5], making early clinical diagnosis challenging. Although nerve conduction studies are considered the gold standard for diagnosing DPN, their time–consuming nature, invasiveness, and limited accessibility impede their widespread application.

Ultrasound (US) has emerged as a noninvasive, cost-effective tool for peripheral nerve evaluation. Conventional grayscale imaging has been used to assess structural nerve changes, such as changes in the cross-sectional area (CSA) and thickness-to-width ratios [6, 7]. However, there are variabilities in diagnostic accuracy among physicians with different levels of experience, as conventional US relies on the subjective interpretation of morphological features such as cross-sectional area and echotexture [8, 9].

Shear wave elastography (SWE) is an ultrasound technique that quantitatively measures tissue stiffness through shear wave propagation velocity. It has proven effective in assessing structural and pathological changes in various tissues, including the liver, thyroid, breast, and musculoskeletal system [10–13]. In peripheral nerve imaging, SWE enables quantitative evaluation of nerve elasticity and has demonstrated lower operator dependency and improved reproducibility than conventional grayscale ultrasound [14–17]. This objectivity may particularly benefit junior physicians, who often find it challenging to recognize subtle morphological changes associated with neuropathy.

Previous studies on SWE in diabetic DPN have mainly focused on quantifying tibial nerve stiffness and establishing diagnostic cut–off values to differentiate DPN from non-DPN patients [18]. However, few have examined how SWE influences diagnostic performance across radiologists with different experience levels or its combined application with grayscale ultrasound in practical diagnostic settings.

The present study investigates whether integrating SWE with grayscale ultrasound improves diagnostic accuracy among junior and senior radiologists. Clarifying this relationship may provide a basis for incorporating SWE into clinical workflows and training programs to enhance early detection of DPN.

## Methods

### Study design and participants

**Fig. 1:**
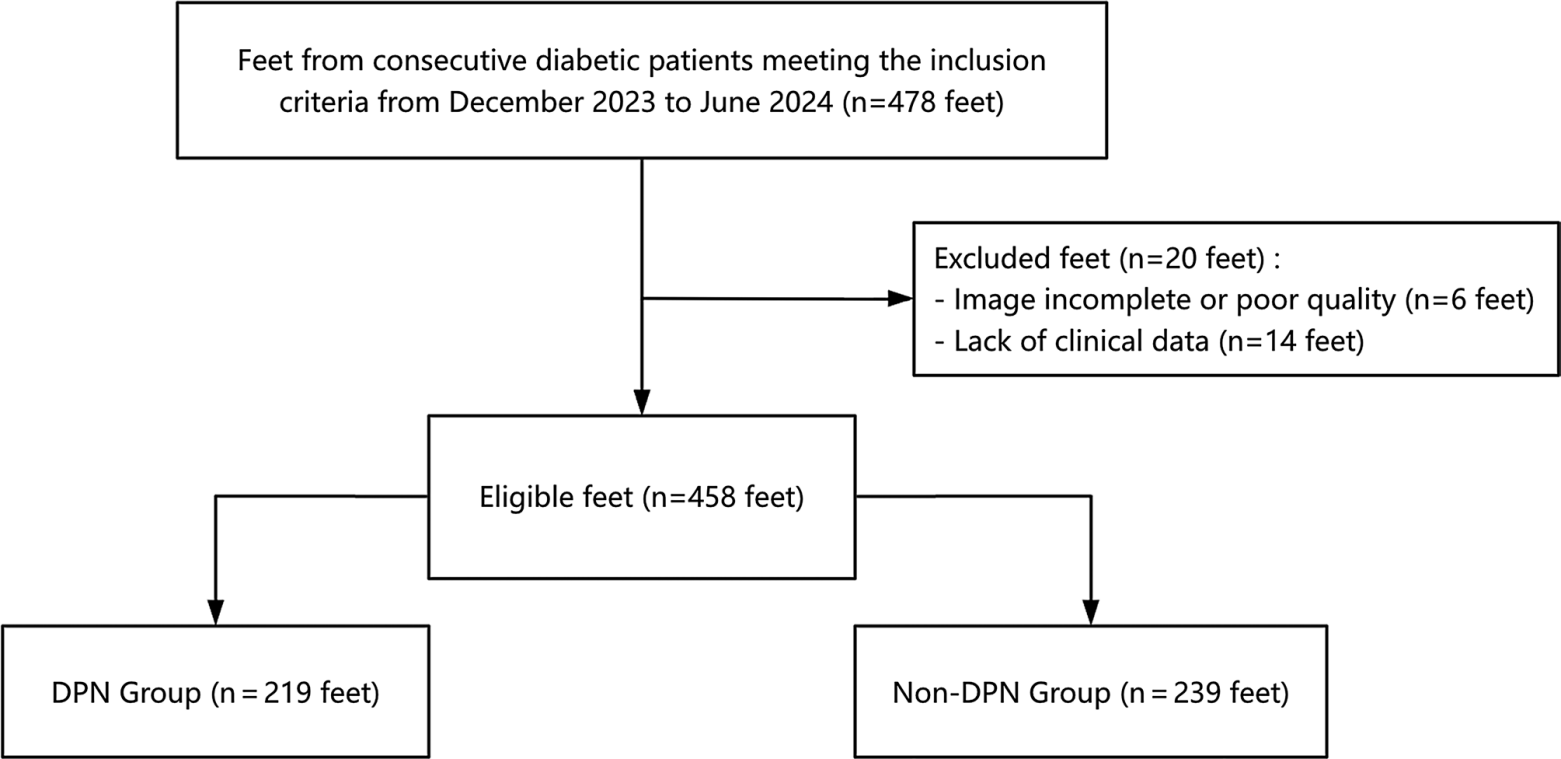
Flowchart of the patient selection. DPN, diabetic peripheral neuropathy; non-DPN, nondiabetic peripheral neuropathy

This study was approved by the Institutional Review Board of Shaoxing People’s Hospital, and informed consent was obtained from all participants. Between December 2023 and June 2024, lower extremity peripheral nerve US examinations were conducted on hospitalized patients diagnosed with T2DM who were willing to participate in the study. The inclusion criteria were as follows: (1) age between 18 and 75 years; (2) diagnosis of T2DM on the basis of World Health Organization criteria (fasting glucose ≥ 7.0 mmol/L, 2–hour post–OGTT glucose ≥ 11.1 mmol/L, or random glucose ≥ 11.1 mmol/L); (3) absence of active diabetic foot ulcers or deformities; (4) no foot trauma or infection due to other causes; and (5) no history of peripheral neuropathy or nerve injury unrelated to diabetes. All patients underwent neuroelectrophysiological evaluation. After 20 patients with incomplete clinical data or suboptimal US image quality were excluded, 458 lower extremities were included in the final analysis (Fig. 1). To account for intra–patient correlation between the two limbs, we performed an analysis using the generalized estimating equation (GEE) model. The baseline demographic and clinical data are summarized in Table 1.

**Table 1:** Baseline characteristics of the study participants

Characteristic	Overall (*n* = 458)	DPN group (*n* = 219)	Non-DPN group (*n* = 239)	*P* Value	FDR-adjusted *P* value
Age (years)	60.00(52.00–70.00)	64.00 (57.00–73.00)	57.00 (48.00–65.00)	< 0.001	< 0.001
Height (m)	161.50 (156.00–169.00)	161.00 (155.00–168.00)	163.50 (156.00–170.00)	0.026	< 0.001
Weight (kg)	64.50 (57.60–73.50)	63.30 (57.40–70.30)	67.20 (58.90–75.60)	0.001	0.002
Sex (male)	244 (53.30%)	116 (53.00%)	128(53.60%)	0.900	0.938
Body mass index (kg/m^2^)	24.87 (22.49–26.89)	24.63 (22.32–26.25)	25.22 (22.95–27.72)	0.015	0.021
Disease duration (months)	120.00 (12.00–180.00)	144.00 (96.00–228.00)	36.00 (4.00–120.00)	< 0.001	< 0.001
HbA1c (%)	8.90 (7.40–11.00)	8.60 (7.30–10.20)	9.30 (7.40–11.20)	0.025	0.035
CRP (mg/L)	1.53 (0.62–3.47)	1.55 (0.68–3.41)	1.45 (0.50–3.83)	0.387	0.426
Systolic blood pressure (mmHg)	132.00 (120.00–144.00)	132.00 (120.00–145.00)	132.00 (120.00–143.00)	0.679	0.738
Diastolic blood pressure (mmHg)	80.00 (73.00–88.00)	77.00 (70.00–85.00)	83.00 (75.00–89.00)	< 0.001	< 0.001
Smoking	163 (35.60%)	78(35.60%)	85(35.60%)	0.991	0.991
Cholesterol (mmol/L)	4.51 (3.92–5.43)	4.35 (3.69–5.22)	4.68 (4.11–5.58)	0.001	0.003
Triglycerides (mmol/L)	1.45 (1.06–2.06)	1.37 (1.05–2.06)	1.59 (1.09–2.06)	0.159	0.209
LDL (mmol/L)	2.70 (2.20–3.28)	2.61 (2.10–3.02)	2.83 (2.32–3.41)	< 0.001	< 0.001
HDL (mmol/L)	0.99 (0.85–1.18)	1.02 (0.85–1.18)	0.98 (0.84–1.19)	0.379	0.440
Creatinine (µmol/L)	64.70 (52.50–76.00)	66.70 (54.10–79.00)	63.50 (50.90–73.90)	0.009	0.015
Urea nitrogen(mmol/L)	5.64 (4.49–6.63)	5.87 (4.64–6.88)	5.35 (4.34–6.32)	0.008	0.013
Fasting blood glucose (mmol/L)	9.08 (7.24–12.65)	8.92 (6.78–12.69)	9.31 (7.27–12.64)	0.215	0.269
TN_MCV (m/s)	43.60 (41.09–46.5)	42.60 (40.77–45.70)	44.30 (42.00–48.00)	< 0.001	< 0.001
SN_SCV (m/s)	49.40 (43.18–56.00)	46.00 (41.10–52.30)	52.30 (46.00–57.50)	< 0.001	< 0.001

Note: Values are presented as median (interquartile range) or number (%)

DPN, diabetic peripheral neuropathy; Non-DPN, non-diabetic peripheral neuropathy; HbA1c, glycated hemoglobin A1c; CRP, C‑reactive protein; LDL, low-density lipoprotein; HDL, high-density lipoprotein; TN-MCV, tibial nerve motor conduction velocity; SN-SCV, sural nerve sensory conduction velocity

*P* < 0.05 was considered statistically significant

### US and SWE protocol

**Fig. 2:**
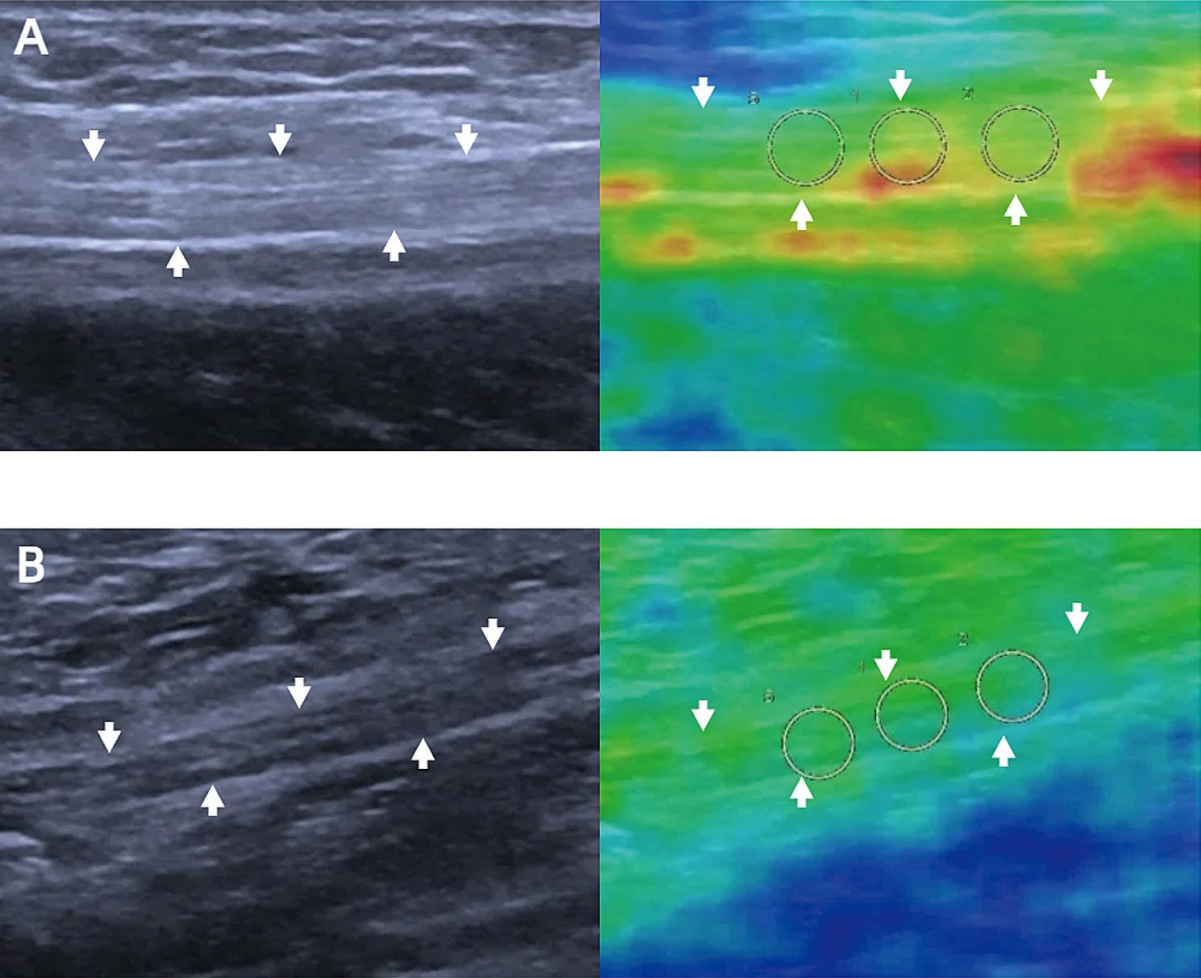
Representative grayscale ultrasound and shear wave elastography images of the tibial nerve (white arrow) (**A**) A 53-year-old DPN patient with shear wave velocities values of 4.43 m/s, 4.24 m/s and 3.49 m/s. (**B**) A 63-year-old non-DPN patient with shear wave velocities values of 2.88 m/s, 2.93 m/s and 3.11 m/s. DPN, diabetic peripheral neuropathy; non-DPN, nondiabetic peripheral neuropathy

All US examinations were performed by a senior sonographer with over 10 years of experience using a Siemens Acuson Sequoia Silver system equipped with a 5–17 MHz high–frequency linear array transducer. After acquisition, all images were anonymized and independently reviewed by four radiologists. Both transverse and longitudinal views of the tibial nerve were obtained approximately 4 cm above the highest point of the medial malleolus. The anteroposterior diameter, transverse diameter, and CSA were measured. The CSA was calculated by manually tracing the outer hyperechoic nerve boundary in the transverse view while minimizing probe compression artifacts. We adopted a standardized data collection protocol to minimize measurement variability. To prevent compression effects, the transducer was positioned lightly on the skin with sufficient coupling gel and remained stationary during the acquisitions. The probe was positioned 4 cm proximal to the medial malleolus and oriented perpendicular to the nerve’s course, avoiding the branch of the tibial nerve to obtain the transverse section of the nerve. The probe was then rotated 90° to obtain a longitudinal section of the tibial nerve. During SWE acquisition, the probe was aligned parallel to the nerve fibers, and minimal pressure was applied to the tissue to ensure consistent contact and reduce anisotropy. The regions of interests (ROIs) were selected on the long–axis plane and placed on the central portion of the targeted nerve, avoiding surrounding tissues such as muscle or fat. Three ROI sizes were standardized to the circle with a diameter of 3 mm, from which both the shear wave velocities and elastic modulus values were measured three times, and the average values were recorded for analysis (Fig. 2). All ROIs maintained identical dimensions.

### Image interpretation

Four independent radiologists, blinded to the clinical data and patient diagnoses, interpreted the anonymized US and SWE images. They were grouped into two experience levels: senior (radiologist 1 and radiologist 2, both with more than 10 years of musculoskeletal US experience) and junior (radiologist 3 and radiologist 4, both with no more than 2 years of musculoskeletal US experience). All images were randomly displayed, and diagnostic assessments were performed under three different conditions: (1) grayscale US alone: evaluation based solely on grayscale US images via a standardized scoring system assessing nerve thickness, internal architecture, echogenicity, and perineural boundaries; (2) SWE alone: diagnosis of DPN was based on the average shear wave velocities; (3) US + SWE: both grayscale US and SWE findings were assessed together, and diagnostic predictions were synthesized via logistic regression modelling. Each method’s results were recorded independently by all four radiologists.

### The intraclass correlation coefficient (ICC) analysis

The ICC analysis was employed to assess both intra-observer and inter-observer repeatability, using a two-way mixed-effects model with absolute agreement for average measurements [ICC (3, k)]. Inter-observer consistency was evaluated based on the predicted probabilities derived from the combined method, independently obtained by junior and senior radiologists. Intra-observer consistency was assessed through repeated evaluations conducted by the same radiologists after a one-month interval.

### Statistical analysis

All the statistical analyses were performed via IBM SPSS Statistics (version 26.0), R software (version 4.5.1), and MedCalc Statistical Software (version 22.001). Normality of continuous variables was assessed using the Kolmogorov–Smirnov test. Continuous variables are expressed as the means ± standard deviations if normally distributed or as medians (interquartile ranges) if not normally distributed. Intergroup comparisons were conducted via independent–sample t tests or Mann–Whitney U tests, as appropriate. Categorical variables are expressed as counts (percentages) and were compared via chi–square tests. To evaluate the potential impact of multiple testing, a Benjamini–Hochberg false discovery rate (FDR) correction was applied. Multivariate logistic regression analysis was conducted to identify independent predictors of DPN. Variables with *P* < 0.05 in univariate analysis were included in the multivariate model. The Hosmer-Lemeshow test was used to evaluate the goodness of fit of the logistic regression model. Diagnostic performance metrics—including sensitivity, specificity, area under the receiver operating characteristic curve (AUROC), positive/negative predictive values (PPV/NPV), and likelihood ratios (LR+/LR−)—were calculated for each radiologist and for each diagnostic method. Sensitivity at 80% specificity for continuous variables was estimated using linear interpolation on the ROC curve. DeLong’s test compared each radiologist’s ROC curves with the combined model. Simple linear regression was used to compare the diagnostic performance of radiologists in different groups with each diagnostic method. ICC was used to assess intra– and inter– observer reproducibility afterward. A two-tailed P value < 0.05 was considered statistically significant.

## Results

### Baseline information of the study population

A total of 458 lower extremities from 236 patients were assessed, comprising 219 lower extremities with clinically confirmed DPN and 239 lower extremities without DPN. As shown in Table 1, there were no significant differences between the groups in terms of sex distribution; systolic blood pressure; smoking status; C‑reactive protein (CRP); or High-Density Lipoprotein (HDL), triglyceride, or fasting glucose levels (*P* > 0.05 for all). However, patients in the DPN group were significantly older [64 (57–73) vs. 57 (48–65) years, *P* < 0.001] and had a longer diabetes duration [144 (96–228) vs. 36 (4–120) months, *P* < 0.001]. Serum creatinine and urea nitrogen were also elevated in the DPN group [66.70 (54.10–79.00) vs. 63.50 (50.90–73.90) µmol/L, *P* = 0.009 and 5.87 (4.64–6.88) vs. 5.35 (4.34–6.32) mmol/L, *P* = 0.008, respectively], suggesting renal involvement. Interestingly, patients without DPN had significantly higher BMI, diastolic pressure, cholesterol, LDL, and HbA1c levels [25.22 (22.95–27.72) vs. 24.63 (22.32–26.25), 83 (75–89) vs. 77 (70–85) mmHg, 4.68 (4.11–5.58) vs. 4.35 (3.69–5.22) mmol/L, 2.83 (2.32–3.41) vs. 2.61 (2.10–3.02) mmol/L, and 9.30 (7.40–11.20) vs. 8.60 (7.30–10.20) %, respectively, *P* values all < 0.05]. To address potential confounders, we performed an additional multivariate logistic regression analysis. The results demonstrated that SWE value remained an independent predictor of DPN status (β = 0.967, OR = 2.629, 95% CI: 1.858–3.720, *P* < 0.001). Meanwhile, age, diabetes duration, and CRP showed a weaker association with DPN status (OR = 1.024, 95% CI: 1.003–1.046; OR = 1.008, 95% CI: 1.006–1.011; OR = 1.017, 95% CI: 1.003–1.031; *P* < 0.05 for all) (Supplementary Table S4 and S5). Additionally, we have verified that the main results remained significant after applying an FDR correction.

As both lower extremities were examined in our study, except in patients with trauma or active ulcers that excluded them from participation, potential intra-subject correlation may exist. Nevertheless, an analysis conducted using the GEE model revealed no significant difference between single-limb and bilateral SWE measurements (*P* = 0.285, CI: -0.061–0.208) in our study, suggesting that this potential dependency did not materially affect our conclusions.

### US and SWE findings

As detailed in Table 2, tibial nerve parameters were significantly greater in the DPN group than in the non-DPN group [transverse diameter: 5.00 (4.60–5.40) vs. 4.20 (3.90–4.40) mm; anteroposterior diameter: 3.10 (2.80–3.40) vs. 2.70 (2.40–2.90) mm; and CSA: 0.10 (0.09–0.12) vs. 0.08 (0.06–0.09) cm²; *P* values all < 0.001]. Significant variations in sonographic characteristics between the DPN and non-DPN groups were consistently noted across all radiologists. Notably, indistinct internal structures and changes in internal echoes were more frequently identified in the DPN group than in the control group by all observers. For example, senior radiologist 1 reported indistinct internal structures in 78.5% of DPN patients compared with 45.5% of non-DPN patients (*P* < 0.001), whereas senior radiologist 2 reported similar findings (66.2% vs. 26.8%, *P* < 0.001). However, radiologist 2 reported no significant difference in the epineurium between the DPN group and the non-DPN group (*P* = 0.172). Additionally, junior radiologists did not observe a significant difference in nerve thickness between the two groups (*P* = 0.084 for junior radiologist 3 and *P* = 0.129 for junior radiologist 4). The comprehensive results from all the observers are detailed in Table S1. SWE measurements revealed markedly increased tibial nerve stiffness in DPN patients [37.30 (29.73–48.87) kPa vs. 25.40 (19.23–33.03) kPa, *P* < 0.001], with corresponding velocities of 3.54 (3.12–4.02) m/s vs. 2.90 (2.53–3.31) m/s.

**Table 2:** Ultrasound features of tibial nerve in different groups

Characteristic	Overall (*n* = 458)	DPN group (*n* = 219)	Non-DPN group (*n* = 239)	*P* Value
TN-TD (mm)	4.50 (4.00–5.10)	5.00 (4.60–5.40)	4.20 (3.90–4.40)	< 0.001
TN-APD (mm)	2.90 (2.60–3.20)	3.10 (2.80–3.40)	2.70 (2.40–2.90)	< 0.001
TN-CSA (cm^2^)	0.09 (0.07–0.10)	0.10 (0.09–0.12)	0.08 (0.06–0.09)	< 0.001
Shear Wave Velocities (m/s)	3.20 (2.76–3.66)	3.54 (3.12–4.02)	2.90 (2.53–3.31)	< 0.001
Elastic Modulus (kPa)	31.02 (23.07–40.12)	37.30 (29.73–48.87)	25.40 (19.23–33.03)	< 0.001

Note: Values are presented as median (interquartile range)

TN-TD, tibial nerve transverse diameter; TN-APD, tibial nerve anteroposterior diameter; TN-CSA, tibial nerve cross-sectional area

*P* < 0.05 was considered statistically significant

### Diagnostic performance of different imaging modalities

Table 3 presents the diagnostic performance of four independent radiologists using different image modalities. For SWE alone, the AUROC was 0.78 (95% CI: 0.74–0.81), with a sensitivity and specificity of 47.95% and 92.89%, respectively. The optimal cut-off value was 3.59 m/s (CI: 3.31–3.66 m/s), which was determined via Youden’s index.

**Table 3:** Diagnostic performance of four independent physicians in detecting DPN before and after combining SWE

Group	AUROC	SEN (%)	SPE (%)	LR+	LR–	PPV (%)	NPV (%)
SWE alone	0.78 (0.74–0.81)	47.95 (41.17–54.78)	92.89 (88.86–95.80)	6.74 (4.18–10.88)	0.56 (0.49–0.64)	86.10 (79.29–90.89)	66.10 (63.06–68.96)
Senior radiologist 1							
US alone	0.68 (0.64–0.72)	78.54 (72.50–83.78)	57.74 (51.20–64.08)	1.85 (1.58–2.19)	0.37 (0.28–0.49)	63.00 (59.12–66.73)	74.59 (69.03–79.46)
US + SWE	0.81 (0.77–0.85)	83.56 (77.98–88.21)	65.27 (58.87–71.29)	2.41 (2.00–2.89)	0.25 (0.18–0.34)	68.80 (64.73–72.59)	81.25 (76.01–85.56)
Senior radiologist 2							
US alone	0.71 (0.67–0.75)	66.21 (59.53–72.44)	75.73 (69.79–81.03)	2.73 (2.14–3.48)	0.45 (0.37–0.54)	71.42 (66.22–76.12)	70.98 (66.72–74.90)
US + SWE	0.83 (0.80–0.87)	83.56 (77.98–88.21)	73.64 (67.57–79.11)	3.17 (2.54–3.95)	0.22 (0.16–0.30)	74.39 (69.98–78.35)	83.02 (78.23–86.93)
Junior radiologist 3							
US alone	0.58 (0.53–0.63)	41.10 (34.51–47.92)	74.90 (68.90–80.26)	1.64 (1.25–2.15)	0.79 (0.69–0.90)	60.00 (53.37–66.28)	58.12 (54.85–61.31)
US + SWE	0.79 (0.75–0.82)	66.21 (59.53–72.44)	82.85 (77.46–87.40)	3.86 (2.88–5.18)	0.41 (0.34–0.50)	77.99 (72.50–82.60)	72.79 (68.78–76.47)
Junior radiologist 4							
US alone	0.60 (0.56–0.65)	38.36 (31.88–45.15)	82.43 (76.99–87.03)	2.18 (1.58–3.01)	0.75 (0.67–0.84)	66.67 (59.18–73.40)	59.34 (56.42–62.19)
US + SWE	0.79 (0.75–0.83)	64.38 (57.65–70.72)	84.10 (78.84–88.50)	4.05 (2.98–5.51)	0.42 (0.35–0.51)	78.77 (73.17–83.47)	72.04 (68.14–75.64)

Note: Values are presented as estimate (95% confidence interval)

US, ultrasound; SWE, shear wave elastography; AUROC, area under the receiver operating characteristic curve; SEN, sensitivity; SPN, specificity; LR+, positive likelihood ratio; LR−, negative likelihood ratio; PPV, positive predictive value; NPV, negative predictive value

**Fig. 3:**
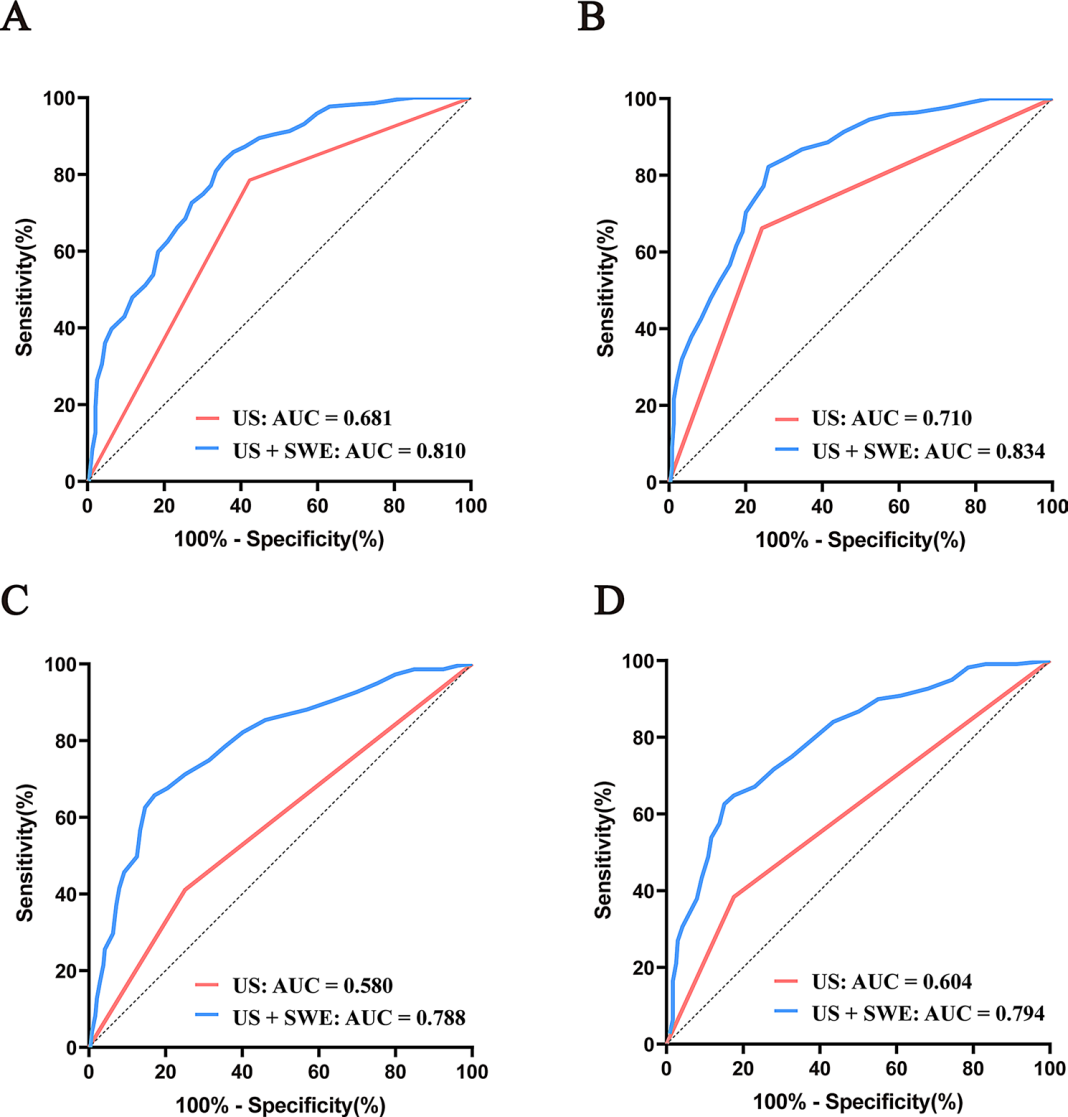
Receiver operating characteristic (ROC) curves for radiologists with different levels of experience (**A**) curve for senior radiologist 1; (**B**) curve for senior radiologist 2; (**C**) curve for junior radiologist 3; (**D**) curves for junior radiologist 4. Each panel compares the performance before and after applying the combined ultrasound elastography method. AUC, area under the curve; SWE, shear wave elastography

**Fig. 4:**
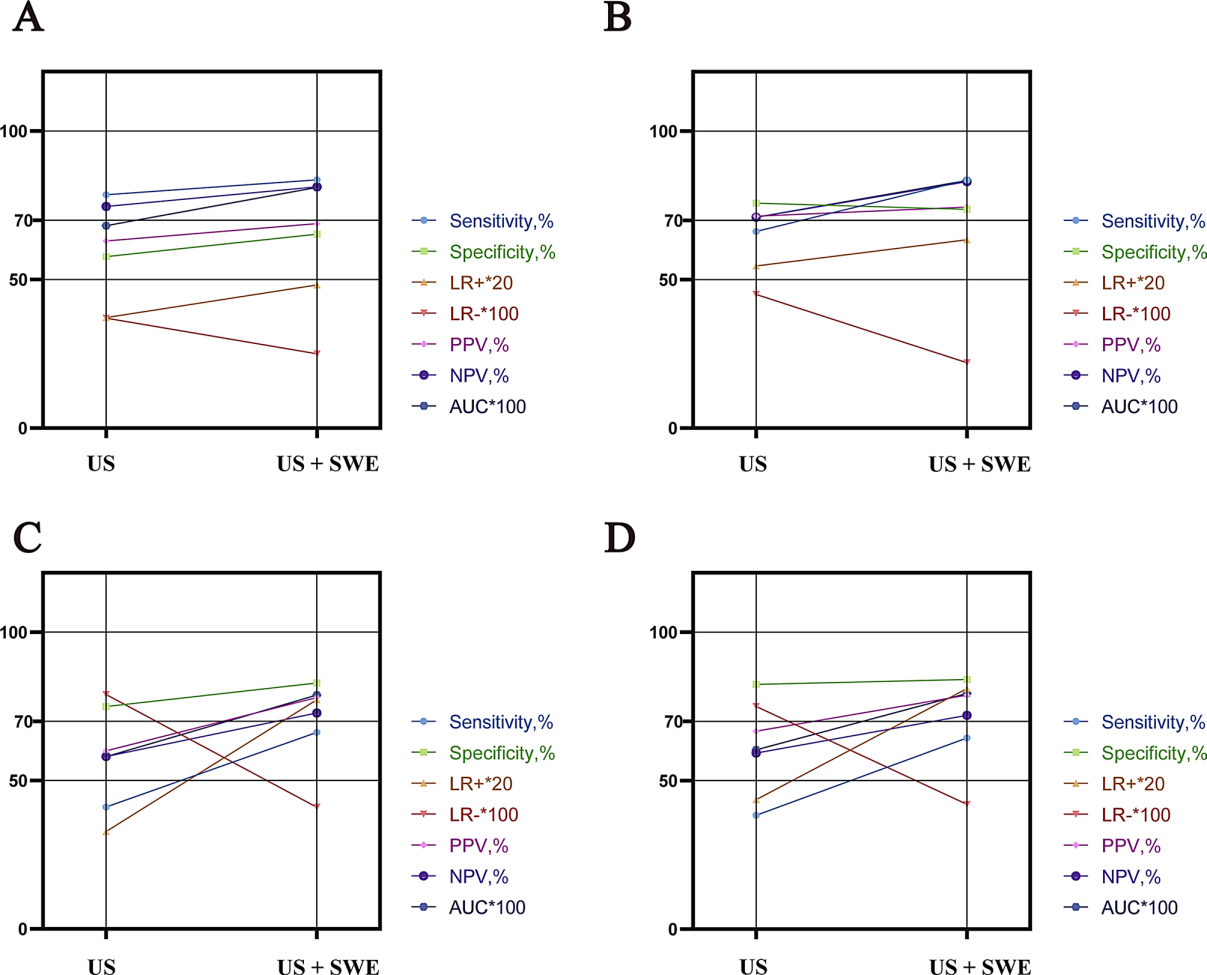
Slope plots of diagnostic efficacy for radiologists with different levels of experience (**A**) Plots for senior radiologist 1; (**B**) plots for senior radiologist 2; (**C**) plots for junior radiologist 3; (**D**) plots for junior radiologist 4. AUC, area under the curve; LR+, positive likelihood ratio; LR−, negative likelihood ratio; PPV, positive predictive value; NPV, negative predictive value

For radiologist 1, combining methods increased the AUROC from 0.68 (95% CI: 0.64–0.72) to 0.81 (95% CI: 0.77–0.85) (*P* < 0.001), improving both the sensitivity (78.54% to 83.56%) and specificity (57.74% to 65.27%). For radiologist 2, the AUROC improved from 0.71(95% CI: 0.67–0.75) with US alone to 0.83 (95% CI: 0.80–0.87) after combining US with SWE (*P* < 0.001). The specificity remained stable, whereas the sensitivity increased from 66.21% (95% CI: 59.53%–72.44%) to 83.56% (95% CI: 77.98%–88.21%). The AUROC of radiologist 3 increased from 0.58 (95% CI: 0.53–0.63) (US alone) to 0.79 (95% CI: 0.75–0.82) (combined) (*P* < 0.001), and the sensitivity increased from 41.10% (95% CI: 34.51%–47.92%)to 66.21%(95% CI: 59.53%–72.44%). The AUROC of Radiologist 4 increased from 0.60 (95% CI: 0.56–0.65) to 0.79 (95% CI: 0.75–0.83) (*P* < 0.001), with the sensitivity improving from 38.36% (95% CI: 31.88%–45.15%) to 64.38% (95% CI: 57.65%–70.72%). For both junior doctors, the specificity remained stable, but the improvement in sensitivity was pronounced (Figs. 3 and 4).

### Comparative analysis between junior and senior radiologists

According to DeLong’s test, each radiologist achieved a higher AUROC with SWE information included. Meanwhile, according to the linear regression analysis, for junior radiologists, the increase in specificity following the application of the combined method was not statistically significant (ΔSpecificity = 4.810, 95% CI: -11.611–21.231, *P* = 0.335). However, the improvement in sensitivity was statistically significant compared with the use of US alone (ΔSensitivity = 25.565, 95% CI: 18.477–32.653, *P* = 0.004) (Table S2). In contrast, for senior radiologists, neither sensitivity nor specificity significantly increased with the integration SWE (ΔSpecificity = 2.720, 95% CI: -39.966–45.406, *P* = 0.810, and ΔSensitivity = 11.185, 95% CI: -15.341–37.711, *P* = 0.211, respectively), as detailed in Table S3.

When the specificity was set at 80%, the resulting sensitivity values were presented in Table S6. Upon comparing sensitivities at this fixed specificity, radiologist 1’s sensitivity improved from 37.20% to 62.10% with the addition of SWE (*P* < 0.001). Similarly, radiologist 2’s sensitivity increased from 54.60% to 68.80% under the same conditions (*P* < 0.001). For radiologist 3, the sensitivity rose from 32.70% to 67.50% (*P* < 0.001). Additionally, radiologist 4 experienced an increase in sensitivity from 40.20% to 66.20% (*P* < 0.001).

### ICC analysis

As demonstrated in Table S7, the intra-group consistency of diagnostic performance was high among both senior physicians [ICC = 0.917 (95% CI: 0.872–0.949, *P* < 0.001)] and junior physicians [ICC = 0.937 (95% CI: 0.902–0.961, *P* < 0.001)]. Additionally, a relatively high level of consistency was observed between radiologists with varying levels of experience, as indicated by an ICC of 0.812 (95% CI: 0.669–0.894, *P* < 0.001).

## Discussion

This study investigated the diagnostic value of combining conventional grayscale US with SWE in the evaluation of DPN, with a particular focus on how this combination affects diagnostic performance across radiologists of different seniority. Our findings suggest that this multimodal imaging approach not only enhances overall diagnostic accuracy but is also particularly beneficial for less experienced radiologists, helping to narrow the diagnostic gap between junior and senior practitioners.

The disparities observed between the DPN and non-DPN groups may reflect distinct underlying pathophysiological mechanisms. Patients in the DPN group were older and had a significantly longer duration of diabetes, which is consistent with the well–established relationship between chronic hyperglycemia, microvascular dysfunction, and peripheral nerve degeneration. These clinical characteristics align with prior research underscoring the role of prolonged metabolic dysregulation in DPN pathogenesis [19]. Elevated levels of creatinine and urea nitrogen in the DPN group also suggest a greater burden of systemic microvascular disease, possibly indicating comorbid diabetic nephropathy [20]. Interestingly, the non-DPN group presented higher BMI, LDL, and HbA1c levels—factors that traditionally signal poorer metabolic control. However, previous studies have shown weak or inconsistent associations between glycemic control (e.g., HbA1c levels) and structural nerve changes, indicating that glycemic burden alone may not fully account for DPN development [21, 22].

US has emerged as a noninvasive tool for the evaluation of peripheral neuropathies, including carpal tunnel syndrome and chronic inflammatory demyelinating polyneuropathy [1]. Traditionally, grayscale US parameters such as the CSA and nerve diameter have been used to assess structural nerve abnormalities. Our study confirmed that patients with DPN exhibit significantly greater transverse and anteroposterior tibial nerve diameters, as well as larger CSA, which is likely due to endoneurial edema, axonal degeneration, and disruption of the blood‒nerve barrier caused by prolonged hyperglycemia [23, 24]. However, the diagnostic accuracy of grayscale US remains moderate, with performance heavily dependent on operator experience. This was evident in our cohort, where senior radiologists outperformed their junior counterparts when US alone was used.

The integration of SWE, which quantitatively assesses tissue stiffness by measuring shear wave velocity, substantially enhanced diagnostic performance. Our results revealed that the shear wave velocity of the tibial nerve was significantly greater in DPN patients, with an optimal cut–off value of 3.59 m/s, yielding a sensitivity of 47.95% and a specificity of 92.89%. These findings are consistent with the ability of the SWE to detect biomechanical changes such as perineurial fibrosis and axonal injury, which may not be apparent on grayscale imaging alone [25, 26]. Previous research has utilized the elastic modulus as a metric for assessing DPN, with diagnostic cut–off values ranging from 45.7 kPa to 68.5 kPa [18, 27, 28]. However, recent guidelines indicate that the elastic modulus is calculated from the shear wave velocities measured by an instrument on the basis of the assumption that the medium behaves as a uniform isotropic elastomer [29]. This assumption is not valid for tissues such as musculoskeletal and nerve tissues, which exhibit significant anisotropic properties. Additionally, the conversion to the elastic modulus results in an increased order of magnitude, thereby amplifying measurement variability and potentially leading to inaccuracies in representing neural stiffness. Consequently, the shear wave velocities is deemed a more appropriate evaluation criterion than the elastic modulus. Notably, the critical shear wave velocities values reported in previous studies align closely with our findings, such as 3.13 m/s reported by Pradhan et al. and 4.11 m/s reported by He et al. [30, 31].

Importantly, our study demonstrated that the diagnostic benefit of SWE is pronounced among less experienced radiologists. While senior radiologists also experienced improvements in the AUC, the gains in sensitivity and specificity were less marked. When evaluating sensitivities at a fixed specificity of 80%, the combined method consistently demonstrated higher sensitivity compared to SWE alone, for both junior and senior radiologists. This suggests a genuine enhancement in detection performance rather than a compromise between sensitivity and specificity. Additionally, the intra- and inter-observer repeatability indicates that the predicted probabilities remain stable for both senior and junior radiologists, demonstrating high consistency across radiologists with varying levels of experience. This finding underscores the robustness and reliability of the prediction model. Prior studies have demonstrated substantial intra- and inter-observer reliability in the measurement of healthy median nerve elasticity [32]. Such enhanced consistency contributes to improved inter-observer agreement among physicians with diverse experience levels [33]. Given the pivotal role of radiologist expertise in the interpretation of ultrasound images, the integration of SWE may facilitate training, enhance diagnostic proficiency among junior physicians, and promote uniformity in clinical practice.

Several limitations should be acknowledged. First, the study included only patients with type 2 diabetes, limiting generalizability to type 1 diabetes or other clinical settings. Future studies could incorporate a broader diabetic population. Second, only the tibial nerve was evaluated. Although this finding is consistent with prior research, DPN is a diffuse polyneuropathy that affects multiple peripheral nerves, such as the sural and median nerves. Comparative studies of different nerves may offer a more comprehensive diagnostic framework. Additionally, all image acquisitions were performed by a single experienced sonographer. Although several measures were taken to reduce measurement errors, such as aligning the probe along the nerve’s long axis and positioning the ultrasound beam as close to perpendicular to the nerve path as possible, operator-dependent bias may exist and limit the reproducibility. Fifth, our study was limited to a single–center population of the same ethnicity and region, which may be influenced by ethnic differences, the disease spectrum specific to Asian populations, and statistical characteristics. These might influence external applicability. Future multicenter studies involving diverse populations and equipment are warranted to validate our findings. Finally, the absence of longitudinal follow–up precludes assessment of disease progression and prognostic modeling. Future prospective studies incorporating follow–up data could develop prognostic models that can assess the progression of neuropathy and predict long–term outcomes.

## Conclusion

This study demonstrates that integrating SWE with conventional grayscale US significantly enhances the diagnostic performance for DPN, which has the potential to reduce operator dependence, particularly among less experienced radiologists. By improving sensitivity and overall diagnostic accuracy, especially in junior physicians, SWE has the potential to standardize DPN screening and assist less–experienced radiologists.

## Supplementary Information

Below is the link to the electronic supplementary material.

Supplementary Material 1

## Data Availability

The datasets used during the current study are available from the corresponding author (ZZJ) on reasonable request.
