# Supplementary material for: Diagnostic value of shear wave elastography for diabetic peripheral neuropathy: comparison between junior radiologists and senior radiologists
Source: BMC Med Imaging. 2025 Dec 29;25:512. doi: 10.1186/s12880-025-02061-w (PMC12751637; doi:10.1186/s12880-025-02061-w)
Supplement: Supplementary file 1 — Supplementary Material 1 [file 12880_2025_2061_MOESM1_ESM.docx]

**Diagnostic Value of Shear Wave Elastography for Diabetic Peripheral Neuropathy: Comparison between Junior Radiologists and Senior Radiologists**

**Table S1.** Basic ultrasound characteristics of the tibial nerve.

**Table S2.** Comparison of specificity and sensitivity in junior radiologists after combining ultrasound elastography, based on linear regression analysis.

**Table S3.** Comparison of specificity and sensitivity in senior radiologists after combining ultrasound elastography, based on linear regression analysis.

**Table S4.** Univariate logistic regression analysis of potential risk factors associated with DPN

**Table S5.** Multivariable logistic regression analysis of factors associated with DPN.

**Table S6.** Sensitivity at a fixed 80% specificity of four independent physicians in detecting DPN before and after combining SWE.

**Table S7.** Intra- and inter-observer reliability of diagnostic performance assessed by the ICC

**Table S1.** Basic ultrasound characteristics of the tibial nerve.

|  |  | DPN group  (n = 219) | Non-DPN group  (n=239) | *P* |
| --- | --- | --- | --- | --- |
| Senior radiologist 1 | |  |  |  |
|  | Nerve Thickness |  |  | **< 0.001** |
|  | Normal | 70 (32.00%) | 188 (78.70%) |  |
|  | Thickening | 149 (68.00%) | 51 (21.30%) |  |
|  | Internal Structure |  |  | **< 0.001** |
|  | Uncertain | 0 (0.00%) | 1 (0.40%) |  |
|  | Distinct | 47 (21.50%) | 130 (54.40%) |  |
|  | Indistinct | 172 (78.50%) | 108 (45.20%) |  |
|  | Internal Echo |  |  | **0.012** |
|  | Uncertain | 0 (0.00%) | 0 (0.00%) |  |
|  | Normal | 116 (53.00%) | 155 (64.90%) |  |
|  | Hypoechoic | 22 (10.00%) | 11 (4.60%) |  |
|  | Hyperechoic | 81 (37.00%) | 73 (30.50%) |  |
|  | Epineurium |  |  | **< 0.001** |
|  | Normal | 102 (46.60%) | 170 (71.10%) |  |
|  | Thickening | 117 (53.40%) | 69 (28.90%) |  |
| Senior radiologist 2 | |  |  |  |
|  | Nerve Thickness |  |  | **< 0.001** |
|  | Normal | 79 (36.10%) | 196 (82.00%) |  |
|  | Thickening | 140 (63.90%) | 43 (18.00%) |  |
|  | Internal Structure |  |  | **< 0.001** |
|  | Uncertain | 0 (0.00%) | 0 (0.00%) |  |
|  | Distinct | 74 (33.80%) | 175 (73.20%) |  |
|  | Indistinct | 145 (66.20%) | 64 (26.80%) |  |
|  | Internal Echo |  |  | **< 0.001** |
|  | Uncertain | 0 (0%) | 1 (0.40%) |  |
|  | Normal | 73 (33.30%) | 176 (73.60%) |  |
|  | Hypoechoic | 10 (4.60%) | 2 (0.80%) |  |
|  | Hyperechoic | 136 (62.10%) | 60 (25.10%) |  |
|  | Epineurium |  |  | 0.172 |
|  | Normal | 207 (94.50%) | 232 (97.10%) |  |
|  | Thickening | 12 (5.50%) | 7 (2.90%) |  |
| Junior radiologist 3 | |  |  |  |
|  | Nerve Thickness |  |  | 0.084 |
|  | Normal | 168 (76.70%) | 197 (82.40%) |  |
|  | Thickening | 51 (23.30%) | 42 (16.70%) |  |
|  | Internal Structure |  |  | **0.020** |
|  | Uncertain | 3 (1.40%) | 4 (1.70%) |  |
|  | Distinct | 133 (60.70%) | 197 (82.40%) |  |
|  | Indistinct | 83 (37.90%) | 38 (15.90%) |  |
|  | Internal Echo |  |  | **< 0.001** |
|  | Uncertain | 3 (1.40%) | 0 (0.00%) |  |
|  | Normal | 149 (68.00%) | 208 (87.00%) |  |
|  | Hypoechoic | 33 (15.10%) | 27 (11.30%) |  |
|  | Hyperechoic | 34 (15.50%) | 4 (1.70%) |  |
|  | Epineurium |  |  | **< 0.001** |
|  | Normal | 175 (79.90%) | 224 (93.70%) |  |
|  | Thickening | 44 (20.10%) | 15 (6.30%) |  |
|  |  |  |  |  |
| Junior radiologist 4 | |  |  |  |
|  | Nerve Thickness |  |  | 0.129 |
|  | Normal | 147 (67.10%) | 178 (74.50%) |  |
|  | Thickening | 72 (32.90%) | 61 (25.50%) |  |
|  | Internal Structure |  |  | **< 0.001** |
|  | Uncertain | 0 (0.00%) | 1 (0.40%) |  |
|  | Distinct | 144 (65.80%) | 183 (76.60%) |  |
|  | Indistinct | 75 (34.20%) | 55 (23.00%) |  |
|  | Internal Echo |  |  | **< 0.001** |
|  | Uncertain | 1 (0.50%) | 1 (0.40%) |  |
|  | Normal | 126 (57.50%) | 154 (64.40%) |  |
|  | Hypoechoic | 72 (32.90%) | 84 (35.10%) |  |
|  | Hyperechoic | 20 (9.10%) | 0 (0.00%) |  |
|  | Epineurium |  |  | **< 0.001** |
|  | Normal | 142 (64.80%) | 189 (79.10%) |  |
|  | Thickening | 77 (35.20%) | 50 (20.90%) |  |

Note: DPN, diabetic peripheral neuropathy; Non-DPN, non-diabetic peripheral neuropathy.

*P* < 0.05 was considered statistically significant.

**Table S2.** Comparison of specificity and sensitivity in junior radiologists after combining ultrasound elastography, based on linear regression analysis.

| Predictors | Estimates |  | 95%CI |  | *P* value |  |
| --- | --- | --- | --- | --- | --- | --- |
| **Specificity** | | | | | |  |
| (Intercept) | 78.665 | | 67.053-90.277 | | 0.001 |  |
| After combining SWE | 4.810 | | -11.611-21.231 | | 0.335 |  |
| Observations | 4.000 | |  |  |  |  |
| R²/adjusted R² | 0.443/0.164 | |  |  |  |  |
|  |  | |  |  |  |  |
| **Sensitivity** | | | | | |  |
| (Intercept) | 39.730 | | 34.718-44.742 | | 0.001 |  |
| After combining SWE | 25.565 | | 18.477-32.653 | | **0.004** |  |
| Observations | 4.000 | |  |  |  |  |
| R²/adjusted R² | 0.992/0.988 | |  |  |  |  |

Note: 95% CI, 95% confidence intervals; SWE, Shear wave elastography.

*P* < 0.05 was considered statistically significant.

**Table S3.** Comparison of specificity and sensitivity in senior radiologists after combining ultrasound elastography, based on linear regression analysis.

| Predictors | Estimates |  | 95%CI |  | *P* value |  |
| --- | --- | --- | --- | --- | --- | --- |
| **Specificity** | | | | | |  |
| (Intercept) | 66.735 | | 36.551-96.919 | | 0.011 |  |
| After combining SWE | 2.720 | | -39.966-45.406 | | 0.810 |  |
| Observations | 4.000 | |  |  |  |  |
| R²/adjusted R² | 0.036/-0.446 | |  |  |  |  |
|  |  | |  |  |  |  |
| **Sensitivity** | | | | | |  |
| (Intercept) | 72.375 | | 53.618-91.132 | | 0.004 |  |
| After combining SWE | 11.185 | | -15.341-37.711 | | 0.211 |  |
| Observations | 4.000 | |  |  |  |  |
| R²/adjusted R² | 0.622/0.433 | |  |  |  |  |

Note: 95% CI, 95% confidence intervals; SWE, Shear wave elastography.

*P* < 0.05 was considered statistically significant.

**Table S4.** Univariate logistic regression analysis of potential risk factors associated with DPN

| Variables | β | SE | Z | *P* value | OR (95%CI) |
| --- | --- | --- | --- | --- | --- |
| Age (years) | 0.047 | 0.008 | 5.834 | **0.000** | 1.048 (1.032-1.065) |
| Height (m) | 0.001 | 0.002 | 0.623 | 0.533 | 1.001 (0.998-1.005) |
| Weight (kg) | -0.028 | 0.008 | -3.659 | **0.000** | 0.973 (0.958-0.987) |
| Sex (male) | -0.024 | 0.187 | -0.126 | 0.900 | 0.977 (0.676-1.410) |
| Body mass index (kg/m^2^) | -0.072 | 0.026 | -2.770 | **0.006** | 0.930 (0.884-0.979) |
| Disease duration (months) | 0.010 | 0.001 | 8.311 | **0.000** | 1.010 (1.008-1.013) |
| HbA1c (%) | -0.096 | 0.041 | -2.317 | **0.021** | 0.908 (0.838-0.985) |
| CRP (mg/L) | 0.016 | 0.007 | 2.315 | **0.021** | 1.016 (1.002-1.030) |
| Systolic blood pressure (mmHg) | 0.004 | 0.005 | 0.843 | 0.399 | 1.004 (0.995-1.013) |
| Diastolic blood pressure (mmHg) | -0.035 | 0.009 | -3.902 | **0.000** | 0.966 (0.949-0.983) |
| Smoking | 0.002 | 0.195 | 0.012 | 0.991 | 1.002 (0.683-1.470) |
| Cholesterol (mmol/L) | -0.142 | 0.076 | -1.874 | 0.061 | 0.868 (0.748-1.007) |
| Triglycerides (mmol/L) | -0.049 | 0.066 | -0.732 | 0.464 | 0.953 (0.837-1.085) |
| LDL (mmol/L) | -0.326 | 0.115 | -2.830 | **0.005** | 0.722 (0.576-0.905) |
| HDL (mmol/L) | 0.419 | 0.314 | 1.335 | 0.182 | 1.521 (0.822-2.815) |
| Creatinine (μmol/L) | 0.011 | 0.004 | 2.712 | **0.007** | 1.011 (1.003-1.019) |
| Urea nitrogen (mmol/L) | 0.081 | 0.043 | 1.914 | 0.056 | 1.085 (0.998-1.179) |
| Fasting blood glucose (mmol/L) | -0.019 | 0.023 | -0.794 | 0.427 | 0.982 (0.937-1.028) |
| Shear wave velocity (m/s) | 1.212 | 0.168 | 7.228 | **0.000** | 3.361 (2.420-4.670) |

Note: DPN, diabetic peripheral neuropathy; SE, standard error; Z, statistic; β, regression coefficient; OR, odds ratio; 95% CI, 95% confidence intervals; HbA1c, glycated hemoglobin A1c; CRP, C-reactive protein; LDL, low–density lipoprotein; HDL, high–density lipoprotein.

*P* < 0.05 was considered statistically significant.

**Table S5.** Multivariable logistic regression analysis of factors associated with DPN.

| Variables | β | SE | Z | *P* value | OR (95%CI) |
| --- | --- | --- | --- | --- | --- |
| Age (years) | 0.024 | 0.011 | 2.238 | **0.025** | 1.024 (1.003-1.046) |
| Disease duration (months) | 0.008 | 0.001 | 5.896 | **0.000** | 1.008 (1.006-1.011) |
| Weight (kg) | -0.020 | 0.018 | -1.161 | 0.246 | 0.980 (0.947-1.014) |
| Body mass index (kg/m^2^) | 0.032 | 0.059 | 0.543 | 0.587 | 1.032 (0.920-1.158) |
| Diastolic blood pressure (mmHg) | -0.007 | 0.011 | -0.642 | 0.521 | 0.993 (0.972-1.014) |
| CRP (mg/L) | 0.017 | 0.007 | 2.460 | **0.014** | 1.017 (1.003-1.031) |
| LDL (mmol/L) | 0.040 | 0.139 | 0.289 | 0.772 | 1.041 (0.792-1.368) |
| Creatinine (μmol/L) | 0.005 | 0.005 | 1.109 | 0.267 | 1.005 (0.996-1.015) |
| HbA1c (%) | -0.005 | 0.051 | -0.094 | 0.925 | 0.995 (0.901-1.099) |
| Shear wave velocity (m/s) | 0.967 | 0.177 | 5.460 | **0.000** | 2.629 (1.858-3.720) |

Note: DPN, diabetic peripheral neuropathy; SE, standard error; Z, statistic; β, regression coefficient; OR, odds ratio; 95% CI, 95% confidence intervals; HbA1c, glycated hemoglobin A1c; CRP, C-reactive protein; LDL, low-density lipoprotein.

*P* < 0.05 was considered statistically significant.

**Table S6.** Sensitivity at a fixed 80% specificity of four independent physicians in detecting DPN before and after combining SWE.

|  | AUROC | Sensitivity (%) * | *P* value | |
| --- | --- | --- | --- | --- |
| **SWE alone** | 0.78 (0.74-0.81) | 58.90 |  |  |
| **Senior radiologist 1** |  |  | <0.001 |  |
| US alone | 0.68 (0.64-0.72) | 37.20 |  |  |
| US+SWE | 0.81 (0.77-0.85) | 62.10 |  |  |
| **Senior** **radiologist 2** |  |  | <0.001 |  |
| US alone | 0.71 (0.67-0.75) | 54.60 |  |  |
| US+SWE | 0.83 (0.80-0.87) | 67.90 |  |  |
| **Junior radiologist 3** |  |  | <0.001 |  |
| US alone | 0.58 (0.53-0.63) | 32.70 |  |  |
| US+SWE | 0.79 (0.75-0.82) | 67.60 |  |  |
| **Junior radiologist 4** |  |  | <0.001 |  |
| US alone | 0.60 (0.56-0.65) | 40.20 |  |  |
| US+SWE | 0.79 (0.75-0.83) | 66.20 |  |  |

Note: DPN, diabetic peripheral neuropathy; SWE, shear wave elastography; AUROC, area under the receiver operating characteristic curve; Sensitivity (%) *, sensitivity at a fixed specificity of 80%.

*P* values were obtained from DeLong’s test for comparison of ROC curves between US alone and US+SWE. *P* < 0.05 was considered statistically significant.

**Table S7.** Intra- and inter-observer reliability of diagnostic performance assessed by the ICC

| Group | Comparison Type | ICC (95% CI) |
| --- | --- | --- |
| Senior radiologists | Intra-observer | 0.917 (0.872-0.949) |
| Junior radiologists | Intra-observer | 0.937 (0.902-0.961) |
| Senior vs. Junior radiologists | Inter-observer | 0.812 (0.669-0.894) |

Note: ICC, intraclass correlation coefficient; 95%CI, 95%confidence interval.

ICC was calculated using a two-way mixed-effects model with absolute agreement for average measures [ICC (3, k)]. ICC values were interpreted as follows: < 0.5, poor; 0.5–0.75, moderate; 0.75–0.9, good; and > 0.9, excellent reliability.
